# Study of ZnO-CNT Nanocomposites in High-Pressure Conditions

**DOI:** 10.3390/ma14185330

**Published:** 2021-09-15

**Authors:** Laura-Madalina Cursaru, Sorina Nicoleta Valsan, Maria-Eliza Puscasu, Ioan Albert Tudor, Nicoleta Zarnescu-Ivan, Bogdan Stefan Vasile, Roxana Mioara Piticescu

**Affiliations:** 1 National R&D Institute for Non-Ferrous and Rare Metals, INCDMNR-IMNR, 077145 Pantelimon, Romania; mpopescu@imnr.ro (L.-M.C.); svalsan@imnr.ro (S.N.V.); epuscasu@imnr.ro (M.-E.P.); atudor@imnr.ro (I.A.T.); nicoleta_sim@yahoo.com (N.Z.-I.); 2 National Research Center for Micro and Nanomaterials, University POLITEHNICA of Bucharest, 011061 Bucharest, Romania; bogdan.vasile@upb.ro

**Keywords:** carbon nanotubes, zinc oxide, in situ hydrothermal synthesis, nanocomposites

## Abstract

Recently, carbon nanotubes (CNTs) have been used extensively to develop new materials and devices due to their specific morphology and properties. The reinforcement of different metal oxides such as zinc oxide (ZnO) with CNT develops advanced multifunctional materials with improved properties. Our aim is to obtain ZnO-CNT nanocomposites by in situ hydrothermal method in high-pressure conditions. Various compositions were tested. The structure and morphology of ZnO-CNT nanocomposites were analyzed by Fourier transform infrared spectroscopy (FTIR), differential scanning calorimetry—thermogravimetry (DSC-TG), X-ray diffraction (XRD), scanning electron microscopy (SEM), energy dispersive X-ray spectroscopy (EDX), and transmission electron microscopy (TEM). These analyses showed the formation of complex ZnO-CNT structures. FT-IR spectra suggest possible interactions between CNT and ZnO. DSC-TG analysis also reveals the formation of some physical bonds between ZnO and CNT, through the appearance of endothermic peaks which could be assigned to the decomposition of functional groups of the CNT chain and breaking of the ZnO-CNT bonds. XRD characterization demonstrated the existence of ZnO nanocrystallites with size around 60 nm. The best ZnO:CNT composition was further selected for preliminary investigations of the potential of these nanocomposite powders to be processed as pastes for extrusion-based 3D printing.

## 1. Introduction

Nanostructured composites based on metal oxides such as TiO_2_ or ZnO are widely studied due to their properties which recommends them for different applications.

It is well-known that TiO_2_ nanoparticles have been used as photocatalysts for the decomposition of organic compounds due to their enhanced photocatalytic activity, chemical stability, semiconducting properties, being inexpensive and environmentally friendly material.

On the other hand, ZnO with excellent electronic properties, large band gap energy in the near-UV region (3.37 eV) similar to TiO_2_, high chemical resistance, strong oxidation ability, non-toxicity, low cost, and earth abundancy [1,2,3,4,5,6], exhibits better photocatalytic activity than TiO2 for the decomposition of organic compounds in several cases [4,7,8], when it is obtained as a nanomaterial with a high BET surface area.

ZnO can be considered as a proper and cheaper choice to TiO_2_ for pollutants degradation (wastewater treatment) [9]. One of the main advantages in the usage of zinc oxide nanoparticles in wastewater treatment is the fact that this kind of material is environmental-friendly. ZnO has been used for photocatalytic decomposition of residual dyes in the treatment of textile and paper wastewater [4,10,11,12].

**Figure 1:**
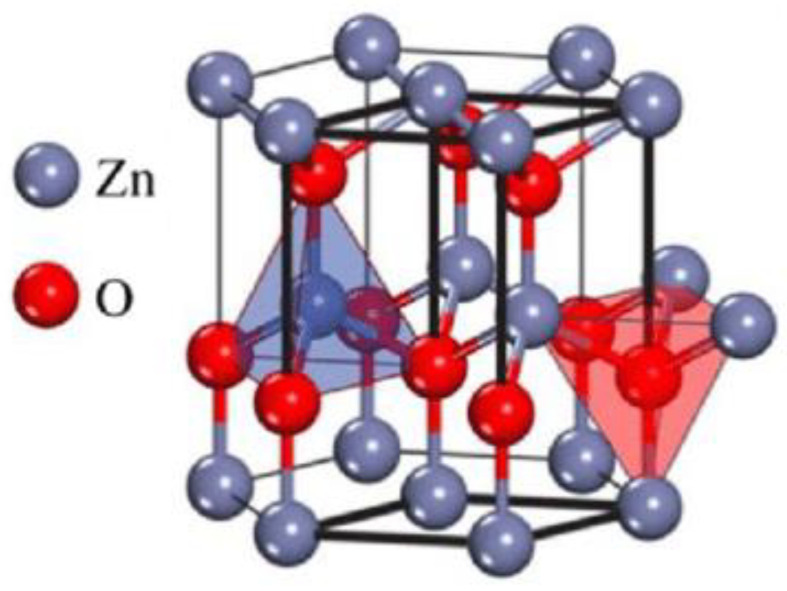
Hexagonal (wurtzite) structure of ZnO [14].

Hexagonal ZnO (wurtzite) presented in Figure 1 can be constructed by considering two interpenetrating lattices similar to II-VI semiconductors. This explains its high efficiency as n-type semiconductor photocatalyst in the UV region [13,14,15,16]. Consequently, ZnO can decompose organic contaminants into CO_2_ and H_2_O in UV light but not in visible light. Therefore, the main drawback of ZnO nanoparticles consists in its reduced ability to use solar energy efficiently. Considering that photodegradation properties of zinc oxide nanoparticles under visible light need to be improved, an interesting strategy could be to combine the ZnO with other semiconductors [17,18,19,20]. Hence, different ZnO-based composite materials have been considered to increase the photocatalytic activity in the visible spectrum.

An intensively studied semiconductor material is represented by carbon nanotubes (CNT). This form of carbon has exceptional structural and electronic properties which can be modified easily by functionalization of the material surface [21]. Carbon nanotubes remain one of the most studied nanomaterials due to its elastic, mechanical, and electrical properties that lead to various applications in energy storage (hydrogen, solar energy), medicine (drug delivery systems), water and wastewater treatment, air pollution control (gas sensor).

Carbon nanotubes can be used in energy storage devices as a result of their high electrical conductivity, high electrolyte accessibility, and stability. CNT presents large specific surface area, fast kinetics, selectivity for aromatic structures, and the capability to remove a great variety of contaminants such as dichlorobenzene, Ni (II), Pb^2+^, Cu^2+^, Cd^2+^, ethyl benzene, dyes, bacteria, viruses, etc., [22,23].

Carbon nanotubes have also been studied for environmental applications due to their distinctive hollow structure, significant electronic, conductive, and physical properties as well as chemical inertness [15,16,24]. CNTs have a mesoporous surface that allows pollutants to be physically adsorbed on [6,25,26].

However, CNT disadvantages represented by its poor dispersion ability and problematic separation process prevent the use of this adsorbent material by itself [18,27,28].

In order to overcome the limitations of the above-mentioned materials, a possible solution can be to obtain a ZnO-CNT-based composite. The photocatalytic activity of zinc oxide combined with the reinforcement and adsorption properties provided by CNT could lead to a material with promising photocatalytic efficiency, surface area and electrical conductivity [29]. Moreover, carbon nanotubes act as a photosensitizer for ZnO improving its photocatalytic performance in the visible light [30]. So far, numerous studies have reported the improved photocatalytic efficiency of ZnO in presence of carbon nanotubes [31,32,33]. The combination of CNT capacitance and metal oxide pseudocapacitive properties can improve the structural and electrochemical features of the materials used in energy storage devices. Thus, ZnO-CNT nanocomposites have a tremendous potential in the fabrication of electrochemical capacitors [34]. In different studies, ZnO nanoparticles successfully bind to the surface of CNTs, as a result of electrons that flow from the conduction band of ZnO to carbon nanotubes surface [35,36].

ZnO-CNT nanocomposite shows many improved mechanical, electrical, and sensing properties that cannot be attained by CNTs and ZnO alone. The nanodevices developed using ZnO-CNT nanocomposite come with improved performance and numerous applications, such as nanoresonators [37], biosensors with high sensitivity and selectivity [38,39,40,41,42] and super capacitors [43]. The studied nanocomposite opens the door to develop new devices with enhanced properties and thus expanding the applications of ZnO and CNT in the field of nanotechnology [44].

Various preparation methods [44] have been used in order to improve the tremendous properties of ZnO-CNTs nanocomposite. Among these, solution-based methods (such as solvothermal decomposition, hydrothermal process, sol-gel hydrothermal method, microwave assisted synthesis [6,9,24,45,46,47,48,49,50,51,52,53,54]), vacuum-based methods (such as reactive magnetron sputtering, atomic layer deposition, filtered cathodic vacuum arc technique, gas-phase deposition, aerosol techniques [19,31,32,55]), and mechanical alloying or mechanical mixing method [44,56] are mentioned.

For example, Wang et al. [51] have prepared CNT-ZnO composites (with 20% and 30% ZnO, respectively) in solution (chemical method) starting from organic precursors. The final products were thermally treated at 400 °C for 2 h to remove the organic part. Dai et al. [48] and Ranjithkumar et al. [53] used chemical reflux method for the synthesis of CNT-ZnO nanocomposites. Azqhandi et al. [24] applied microwave-assisted hydrothermal method to obtain Cd-doped ZnO/CNT nanocomposites. In 2009, Yan et al. [9] investigated hydrothermal synthesis of ZnO-CNT nanocomposite starting from ZnO nanoparticles prepared previously and acid-treated multi-walled carbon nanotubes (MWCNT). In 2006, Zhang et al. [55] have grown ZnO nanowires on modified carbon nanotube arrays by a hydrothermal process. Low amount of acid-treated MWCNT (<1%) have also been incorporated in ZnO by mechanical mixing [56].

The aim of this study is to demonstrate the feasibility of hydrothermal method (a well-known wet chemical synthesis procedure) to develop new types of ZnO-CNT nanocomposite powders in high-pressure conditions for further use in additive manufacturing process (extrusion-based 3D printing) of ZnO-CNT 3D structures.

To our knowledge, no research data regarding fabrication of ZnO-CNT 3D structures by additive manufacturing techniques were found. As a novelty, ZnO-CNT nanocomposite powders resulted from hydrothermal synthesis are further used to test their potential to be processed in the form of pastes for extrusion-based 3D printing.

In the present paper, ZnO-CNT nanocomposites having different ZnO: CNT mass ratios are synthesized in situ by hydrothermal process in high pressure conditions, starting from multi-wall carbon nanotubes (MWCNT) functionalized by acid treatment and water-soluble Zn salt (as ZnO precursor). Zinc oxide is obtained in the presence of MWCNT functionalized with carboxylic groups (MWCNT-COOH), aiming to form a nanocomposite structure with physical bonds between ZnO and MWCNT-COOH. Both functionalized MWCNT and ZnO-CNT nanocomposite powders were characterized by physical, structural, and thermal methods. The best ZnO:CNT composition was selected for preliminary investigations regarding the obtaining of some printable pastes that can be used in additive manufacturing of nanocomposite 3D structures.

## 2. Materials and Methods

MWCNT powder (outer diameter = 10 nm; inner diameter = 4.5 nm; length = 4 μm) was purchased from Sigma-Aldrich, St. Louis, MO, USA; nitric acid p.a., 65% G.R. (HNO_3_) was purchased from LACH-NER, Brno, Czech Republic; sodium hydroxide, reagent grade, ≥98%, was purchased from Lachema, Brno, Czech Republic; sulfuric acid 95–97%, and zinc nitrate hexahydrate, Zn(NO_3_)_2_*6H_2_O reagent grade, 98%, were purchased from Merck, Darmstadt, Germany.

### 2.1. Functionalization of Multi-Walled Carbon Nanotubes (MWCNT)

Multi-walled carbon nanotubes were dispersed in a solution of HNO_3_:H_2_SO_4_ = 1:3 (%vol) and stirred magnetically or ultrasonicated for 8 h. Thus, obtained solutions were centrifuged several times at 6000 rpm, 30 min, until pH = 4–4.5, using a Rotofix 32A centrifuge, Hettich Zentrifugen, Tuttlingen, Germany and then the samples were evaporated under reduced pressure in a Heidolph Rotary Evaporator, Laborota 4000, Schwabach, Germany or lyophilized with an Alpha 1-2 LDplus freeze dryer (Martin Christ Gefriertrocknungsanlagen GmbH, Osterode am Harz, Germany). The resulted CNT powders were subjected to spectral, thermal, and morpho-structural characterizations to demonstrate the functionalization of CNT. The experimental working conditions for the functionalization of MWCNT are shown in Table 1.

### 2.2. Hydrothermal Synthesis of ZnO-CNT Nanocomposites

Nanocomposite materials based on ZnO and functionalized CNT were obtained by hydrothermal process, using as precursors Zn(NO_3_)_2_·6H_2_O, functionalized CNT powder, and NaOH solution 0.5 M. CNT powder functionalized as described above (Section 2.1) was dispersed in NaOH solution 0.5 M, brought to pH = 4–4.5 and ultra-sounded for 15 min, resulting in a CNT solution. Zinc nitrate hexahydrate was dissolved in water and gradually added, under magnetic stirring, to the CNT solution previously heated to 50 °C. Several types of ZnO-CNT nanocomposite powders with different CNT:ZnO weight ratios were prepared (as presented in Table 2).

After precipitation with NaOH solution 0.5 M at pH = 9–9.5, the resulted suspension was poured into the Teflon reaction vessel and introduced in a 1000 mL autoclave (SAM Romania) for hydrothermal synthesis at 200 °C and 100 atm. After hydrothermal treatment, nanocomposite powder was filtered and washed with distilled water up to pH = 7, then dried in oven at 100 °C. ZnO-CNT nanopowders were further investigated by spectral, thermal, morpho-structural, and physical characterization methods in order to find which CNT:ZnO mass ratio is proper for the fabrication of 3D nanocomposite structures.

### 2.3. Spectral, Thermal and Morpho-Structural Characterization of the Prepared Samples

Fourier Transform Infrared Spectroscopy (FT-IR) analysis was accomplished with an ABB MB 3000 FT-IR spectrometer (ABB Inc., Québec, QC, Canada), using the EasiDiff device (PIKE Technologies, Inc., Madison, WI, USA) for powders measurement. The solid composite sample (1% by weight) is mixed with KBr. For data acquisition, 64 scans were run at a resolution of 4 cm^−1^ between 550 and 4000 cm^−1^. All spectra were registered in transmittance mode. Experimental data were processed using the Horizon MB^TM^ FTIR software version 3.4.0.3 (ABB Inc., Québec, QC, Canada).

Differential scanning calorimetry–thermogravimetry (DSC-TG) analysis was performed using the Setaram Setsys Evolution device (Setaram Instrumentation, Caluire, France), in an inert gas atmosphere. Samples were introduced in alumina crucibles and heated up to 800 °C, with heating rate of 10 K/min and cooling rate of 10 K/min. Experimental data were processed with Calisto software v1.097 (Setaram Instrumentation, Caluire, France).

X-ray diffraction (XRD) characterization was carried out with a Bruker-AXS D8 ADVANCE diffractometer Bragg-Brentano diffractometer (Bruker AXS GmbH, Karlsruhe, Germany) with radiation source (Cu) and SOL X vertical geometry detector θ–θ, equipped with BRUKER AXS software. Diffraction spectra were acquired in the angular range 4–74°, continuously, with a step of 0.02°. The phase identification was done with the DIFFRAC.EVA software release 2016 (Bruker AXS GmbH, Karlsruhe, Germany) and the ICDD database PDF 4 + 2020.

Morphological characterization was achieved with a scanning electron microscope SEM Quanta 250 (FEI Company, Eindhoven, The Netherlands) in high vacuum (HV), using the secondary and backscattered electron detector (ETD), and the energy-dispersive detector (EDS).

TEM investigations were made using a high-resolution transmission electron microscope Titan THEMIS (Thermo Fisher Scientific, Waltham, MA, USA-Former FEI), operated at 200 kV. The selected sample was dispersed into a small amount of distilled water, and ultrasonicated for 5 min. After that 10 µL of suspension was placed onto a 400-mesh lacey carbon coated copper grid.

### 2.4. Extrusion-Based 3D Printing of ZnO-CNT Nanocomposite Powders

Preliminary experiments have been performed to prepare proper pastes from ZnO-CNT nanocomposite powders for fabrication of ZnO-CNT 3D structures by extrusion-based 3D printing. Selected nanocomposite powders (sample code CNT-ZnO-2) were mixed with different polymeric additives (dispersants, extrusion agents, viscosity agents) using a Thinky-ARE 250 planetary centrifugal mixer (Thinky Coroporation, Tokyo, Japan) to prepare a homogenous and printable paste, with the removal of air bubbles. The container used for mixing and degassing (defoaming) the paste is the 10 mL cartridge of the 3D-Bioplotter printer. All the prepared pastes were subjected to 2 min of mixing with a speed of 2000 rpm and 2 min of defoaming with a speed of 2000 rpm. ZnO-CNT 3D structures were obtained by extrusion-based 3D printing method with the 3D-BioPlotter Starter system (EnvisionTEC GmbH, Gladbeck, Germany) by dispensing the paste from the 10 mL cartridge through a needle tip of 0.4 mm diameter from a 3-axis system. A printing speed of 8–15 mm/s and a pressure range of 2–4 bars were applied. In order to create the 3D object, square cuboids with dimensions of 10 × 10 × 5 mm were designed with CAD software (SolidWorks 2019, Dassault systems, Waltham, MA, USA). The inner pattern of the designed sample had a line-based hatch type, with continuous strands and a distance between strands of 1.7 mm. The rotation angle between 2 successive layers was 45° and 135°, respectively.

## 3. Results and Discussion

### 3.1. Hydrothermal Synthesis of ZnO-CNT Nanocomposites

The hydrothermal method has several advantages over other methods because it does not use organic or organo-metallic precursors that require subsequent heat treatments (calcination, sintering), it is environmentally friendly because it takes place in closed reaction systems and nano-crystalline powders with controlled size and shape are formed directly from the solution [57,58,59]. It is a relatively cheaper method of synthesis because it requires lower temperatures than other methods (<350 °C).

Unlike Ding et al. [60] who obtained a ternary HA/ZnO/CNT nanocomposite by hydrothermal reaction at 180 °C for 8 h, this paper proposes the application of an external pressure in hydrothermal synthesis conditions, which should favor the physical interactions between zinc oxide and carboxylic groups on the surface of carbon nanotubes.

In this case, the reaction time is shortened to 2 h and the energy consumption is reduced because less energy is needed to apply a pressure of 100 atm into the reaction system than to increase the temperature by a few degrees. According to [61,62] the energy required to increase the temperature by 5 units is the same as that required to increase the pressure by 4000 units.

By applying an external pressure (argon bubbling above the reaction vessel in which the precursor suspension of ZnO-CNT is located), a small compressibility appears in the reaction medium and chemical reactions characterized by a negative ΔV value (ΔV representing the volume difference between reaction products and reactants) take place.

Due to the applied pressure, solubility of the solvated species increases and the distance between these solvated species decreases, so the chemical reactivity improves.

In addition, dissolution/precipitation reactions take place at the liquid/solid interface until thermodynamic equilibrium is reached.

Under pressure conditions, the mechanism of hydrothermal reactions changes, the reaction rate being determined by diffusive processes.

Chemical reaction for hydrothermal synthesis of ZnO synthesis in the presence of functionalized CNT and formation of ZnO-CNT nanocomposite can be written as follows:

(1) $$\mathrm{Zn}{\left({\mathrm{NO}}_{3}\right)}_{2}\cdot 6H_{2}O+2\mathrm{NaOH}=\mathrm{Zn}{\left(\mathrm{OH}\right)}_{2}+2{\mathrm{NaNO}}_{3}+6H_{2}O$$

(2) $$\mathrm{Zn}{\left(\mathrm{OH}\right)}_{2}+\mathrm{MWCNT}-\mathrm{COOH}\overset{\mathrm{hydrothermal}\;\mathrm{synthesis}}{\to }\mathrm{Zn}-O-\mathrm{OOC}-\mathrm{MWCNT}+H_{2}O$$

(3) $$\mathrm{Zn}{\left(\mathrm{OH}\right)}_{2}\overset{precipitation\;/re-dissolving}{\Leftrightarrow }\mathrm{ZnO}+H_{2}O$$

During hydrothermal synthesis, precipitation/re-dissolving reactions of ZnO from Zn(OH)_2_ take place simultaneously with the interactions between ZnO and functionalized carbon nanotubes (denoted as MWCNT-COOH in Equation (2)). The solvated species in the reaction medium are Zn^2+^ (which is surrounded by water molecules) and MWCNT-COO^−^. As explained above, the distance between these species decreases at high pressure and physical interactions between hydroxide ions around Zn and carboxyl groups of carbon nanotubes may occur.

**Figure 2:**
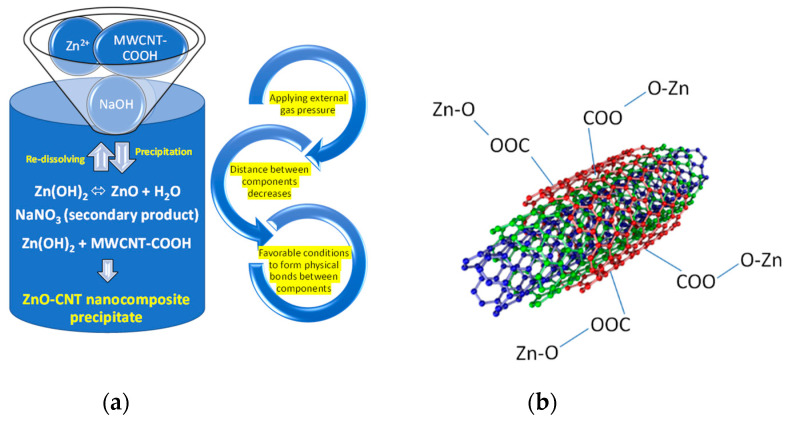
(**a**) The formation of the nanocomposite with physical interactions between the two components (ZnO and MWCNT-COOH); (**b**) possible structure of ZnO-CNT nanocomposite obtained by hydrothermal synthesis in high pressure conditions.

The reactants (Zn^2+^, MW-COOH, NaOH) and the reaction products (ZnO, NaNO_3_, ZnO-CNT) as well as a proposed reaction mechanism for hydrothermal synthesis of ZnO-CNT nanocomposite structure are depicted in Figure 2.

### 3.2. Spectral (FT-IR) Analysis of CNT and ZnO-CNT Samples, Respectively

**Figure 3:**
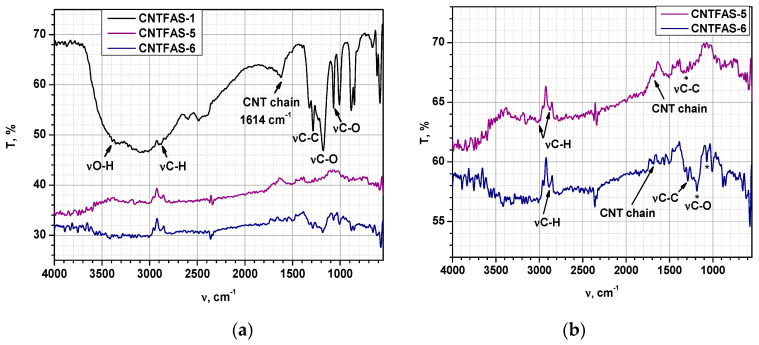
(**a**) FT-IR spectra of CNT samples functionalized by acid treatment. Concentration of HNO_3_ was 4 M in the case of CNTFAS-1 and respectively 2 M in the case of CNFAS-5 and CNTFAS-6 samples. Concentration of H_2_SO_4_ was 10 M for CNTFAS-1 and CNTFAS-5 sample, respectively 5 M for CNTFAS-6 sample; (**b**) detailed FT-IR spectra of CNTFAS-5 and CNTFAS-6 samples.

In order to verify whether the acid treatment of CNT led to the formation of carboxylic groups on the CNT surface, the samples obtained were characterized by FT-IR and DSC-TG. Moreover, the FT-IR analysis may emphasize the formation of ZnO-CNT nanocomposites. FT-IR spectra of the functionalized carbon nanotubes (named CNTFAS-i, as presented in Table 1) are shown in Figure 3. In the case of CNTFAS-1 sample (HNO_3_:H_2_SO_4_ = 1:3 (%vol) mixture of 4 M HNO_3_—10 M H_2_SO_4_), the presence of the following vibration bands is observed: the stretching vibration of the hydroxyl group from COOH (νO-H) at 3437–3466 cm^−1^ [63,64]; deformation vibration of the OH group at 3096–3059 cm^−1^; the stretching vibration of the C-H group at 2953–2889 cm^−1^ [63,64]; vibrations corresponding to carbon nanotube chain at 1614 cm^−1^ [63], C-C bond at 1323–1284 cm^−1^ [63], and the stretching vibration of the C-O group from COOH at 1178 and 1070 cm^−1^ [64]. For all the other investigated samples, dispersion of commercial MWCNT in HNO_3_:H_2_SO_4_ = 1:3 (%vol) mixture of 2 M HNO_3_—10 M H_2_SO_4_, (CNTFAS-5) and HNO_3_:H_2_SO_4_ = 1:3 (%vol) mixture of 2 M HNO_3_—5 M H_2_SO_4_ (CNTFAS-6), respectively, dried by lyophilization, the FT-IR spectra does not reveal the broad peak assigned to hydroxyl groups in the region 3400–3000 cm^−1^. Vibration modes related to CNT could be observed in Figure 3b: C-H stretching vibration appears at 2993, 2878 cm^−1^ (in CNTFAS-5), and 2887 cm^−1^ (in CNTFAS-6); a small shoulder that could be assigned to CNT chain occurs at 1650 cm^−1^ (for CNTFAS-5) and 1684 cm^−1^ for CNTFAS-6. Stretching vibration of C-C bond was detected at 1319 cm^−1^ (CNTFAS-5), 1321, and 1288 cm^−1^ (CNTFAS-6). A sharp peak of C-O bond was also found at 1184 cm^−1^ in the case of CNTFAS-6 sample. Based on these results, it can be considered that CNT functionalization occurs in the following experimental conditions: dispersion of commercial multi-walled carbon nanotubes (MWCNT) in a mixture of HNO_3_ 4 M and H_2_SO_4_ 10 M, with HNO_3_:H_2_SO_4_ = 1:3 (%vol), followed by centrifugation and evaporation under reduced pressure (CNTFAS-1 sample).

**Figure 4:**
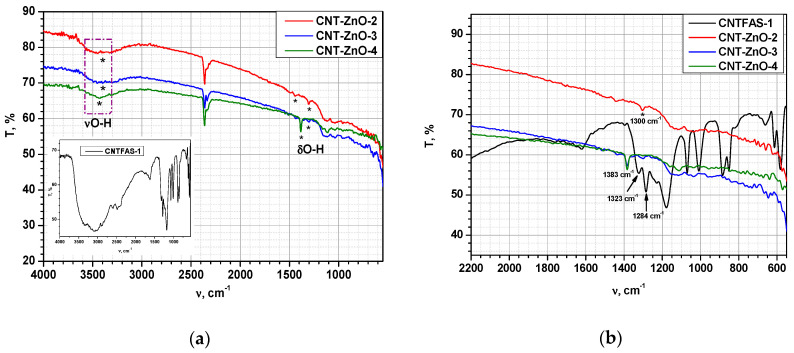
FTIR spectra of ZnO-CNT samples compared to functionalized CNT (CNTFAS-1) in the range of: (**a**) 4000–550 cm^−1^. Inset shows FT-IR spectrum of CNTFAS-1 sample; (**b**) 2000–550 cm^−1^. CNT:ZnO weight ratio for CNT-ZnO-2 = 1:10; for CNT-ZnO-3 = 1:5 and respectively for CNT-ZnO-4 = 1:4.

FT-IR spectra of ZnO-CNT powders described in Table 2 are revealed in Figure 4. A broad peak corresponding to the stretching vibration of the hydroxyl group from COOH (νO-H) is observed at 3389–3398 cm^−1^; vibrations corresponding to the C-H group (around 2900 cm^−1^) and to the carbon nanotube chain (around 1614 cm^−1^), respectively, cannot be observed in the case of nanocomposite powders. These vibrations are probably masked by ZnO which incorporates the whole CNT chain inside its structure; other typical bands assigned to functionalized CNT (at 1284 and 1323 cm^−1^, probably associated to the O-H bending deformation mode of the COOH group) [63,64] are shifted to higher wavenumbers (1300 and 1383 cm^−1^, respectively) in the case of ZnO-CNT nanocomposites. This could be explained by the formation of the structure presented in Figure 2, where some physical bonds between the functional groups of CNT and zinc oxide are expected. Characteristic stretching vibration of metal-oxygen bond (Zn-O in this case) appears at 592 cm^−1^ [65].

### 3.3. Thermal (DSC-TG) Analysis of CNT and ZnO-CNT Samples

**Figure 5:**
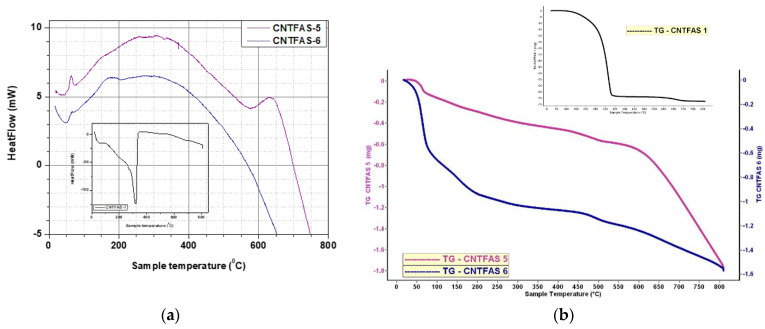
(**a**) DSC-TG curves for: CNTFAS-5 and CNTFAS-6 samples versus CNTFAS-1 sample (inset). (**b**) TG curves for CNTFAS-5 and CNTFAS-6 samples versus CNTFAS-1 sample (inset).

**Figure 6:**
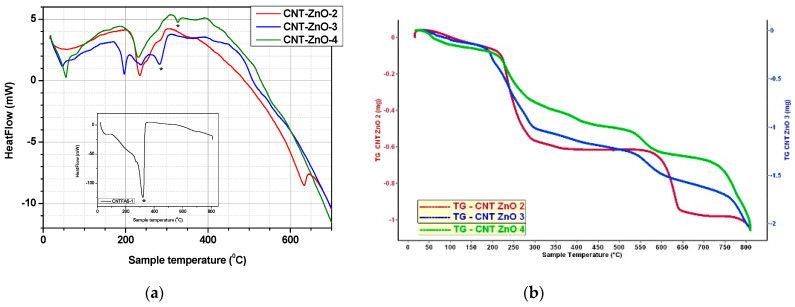
(**a**) DSC-TG curves for: CNT-ZnO samples versus CNTFAS-1 sample (acid mixture 4 M:10 M). CNT:ZnO weight ratio for CNT-ZnO-2 = 1:10; for CNT-ZnO-3 = 1:5 and respectively for CNT-ZnO-4 = 1:4; Inset shows DSC curve of CNTFAS-1 sample (acid mixture 4 M:10 M); (**b**) TG curves forCNT-ZnO-2,CNT-ZnO-3 and CNTZnO-4 samples.

The results obtained at thermal analysis for all CNT samples treated with acid mixtures of various concentrations are presented in Figure 5 and Table 3. Thermal behavior of ZnO-CNT nanocomposites powders and functionalized CNT sample (CNTFAS-1) are presented comparatively in Table 4 and Figure 6. 

DSC-TG curve of CNTFAS-1 sample (shown as a detail in Figure 5), presents a large endothermic peak at 320–322 °C, which could demonstrate CNT functionalization by the presence of carboxyl groups on its surface [66]. As it could be seen in Figure 3, no endothermic peaks corresponding to decomposition of potential carboxylic groups created on the surface of carbon nanotubes are present in CNTFAS-5 and CNTFAS-6 samples, confirming the hypothesis derived from FT-IR results, namely that CNTFAS-1 is the only sample functionalized with carboxylic groups.

In the case of nanocomposite powders, the first endothermic peak is located at 46–63 °C (as presented in Table 4) and could be due to the water evaporation from the surface. It can be observed that ZnO-CNT-based nanocomposite materials are thermally stable up to around 230 °C. A correlation between the total mass loss of nanocomposite samples and CNT content is observed (Table 4), demonstrating the presence of functionalized carbon nanotubes in the nanocomposites structure. The endothermic peaks that appear at 233.7 °C (CNT-ZnO-2), 196.5 °C/231.6 °C (CNT-ZnO-3), and 231.5 °C (CNT-ZnO-4) are probably explained by the breaking of the physical bonds which may appear between carboxylic groups of functionalized MWCNT and ZnO (as shown in Figure 2).

The decomposition of CNT functional groups into CO_2_ can be observed at 290.9 °C in the case of CNT-ZnO-3 (containing 16.7% functionalized CNT) and 326.3°C for CNT-ZnO-4 (containing 20% functionalized CNT), similar to CNTFAS-1 sample (which decomposes at 321.8 °C, as shown in the detail of Figure 6). This could be due to an excess of CNT in the nanocomposite structure.

In the case of CNT-ZnO-2 sample with a content of 9.1% functionalized CNT, no endothermic peak which could be owed to the decomposition of functional groups in the CNT chain was observed. The endothermic peak that appears at 631.7 °C could be explained by the oxidation of MWCNT to CO_2_ [51,67]. This could be explained by a better interaction of CNT with ZnO during hydrothermal synthesis. Therefore, we can consider that CNT-ZnO-2 sample has an optimal composition for the formation of the nanocomposite with physical interactions between the two components.

### 3.4. Morphological Characterization of Functionalised CNT Sample and ZnO-CNT Nanocomposites by Scanning Electron Microscopy (SEM)

**Figure 7:**
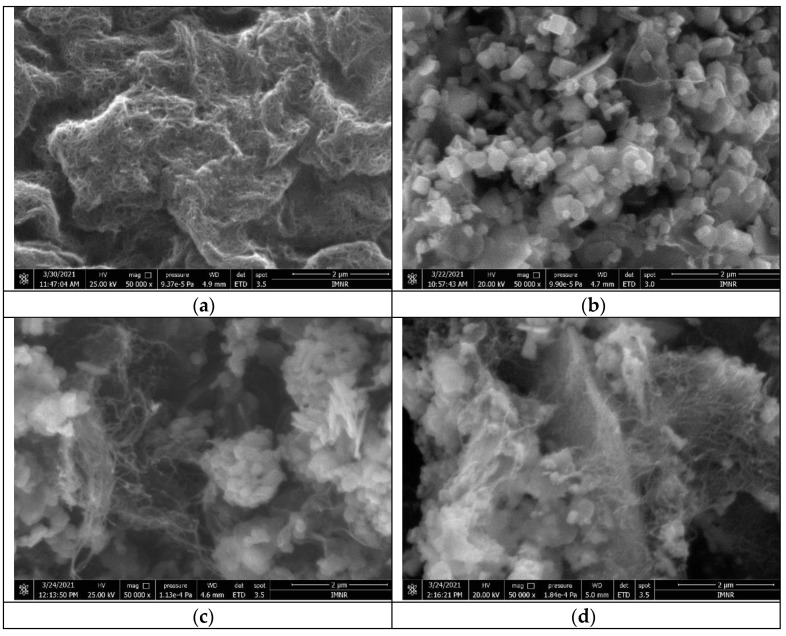
SEM image of: (**a**) CNTFAS -1 sample, 2 µm scale, 50,000× magnification; (**b**) CNT-ZnO-2 sample, 2 µm scale, 50,000× magnification; (**c**) CNT-ZnO-3 sample, 2 µm scale, 50,000× magnification; (**d**) CNT-ZnO-4 sample, 2 µm scale, 50,000× magnification.

SEM image of CNTFAS-1 sample (functionalized CNT) is depicted in Figure 7a, revealing the typical wire morphology of MWCNT. In Figure 7b, it can be observed the existence of polyhedral particles of ZnO, as well as CNT wires, for CNT-ZnO-2 sample (having 9.1% functionalized CNT). CNT wires are better displayed in Figure 7c,d, due to a higher content of carbon nanotubes in ZnO-CNT nanocomposite powders (16.7% and 20%, respectively). In the case of CNT-ZnO-3 sample (Figure 7c) it can be observed that CNT wires are bounded with ZnO particles. It can be seen also large ZnO aggregates having their surface coated with CNT wires (Figure 7d). This is consistent with the results of the thermal analysis, suggesting an excess of CNT (in the case of the CNT-ZnO-4 sample), arranged over the entire ZnO surface.

Characterization of nanocomposite samples by energy dispersive X-ray spectroscopy (EDS) showed the presence of C, O, Zn, and S.

Based on the morphological characteristics of ZnO-CNT nanocomposite powders, it can be concluded that sample CNT-ZnO-2 has a uniform aspect which recommends it for further testing in the preparation of pastes for extrusion-based 3D printing, knowing that particles with a uniform distribution, not agglomerated are suitable for extrusion processing.

### 3.5. Transmission Electron Microscopy (TEM) Characterization of ZnO-CNT Nanocomposite Powder

**Figure 8:**
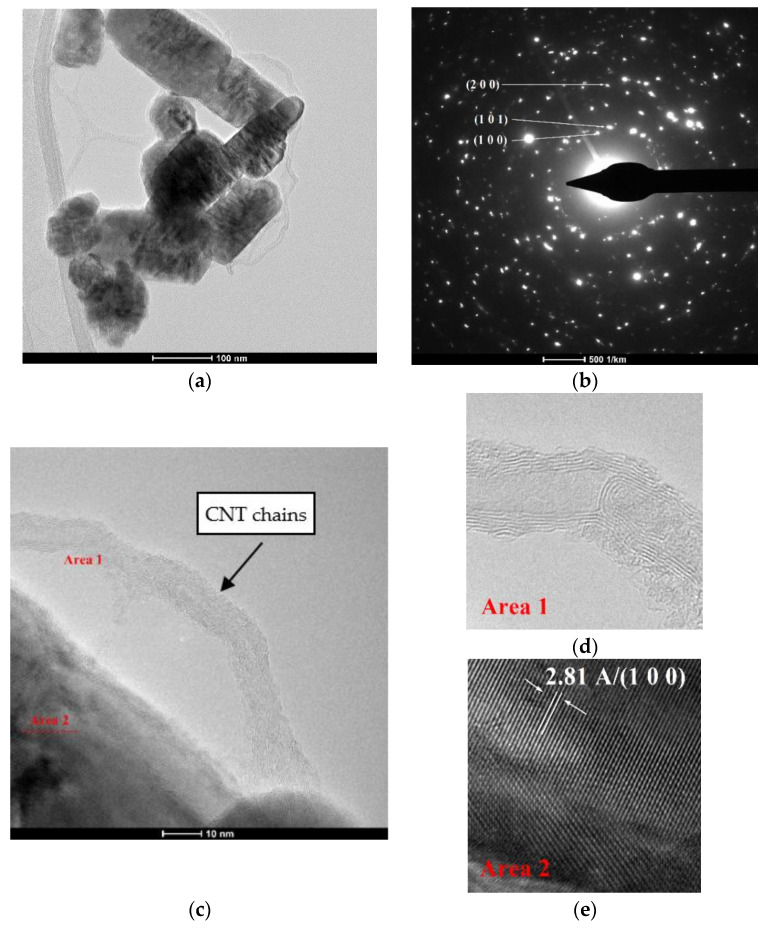
TEM images of CNT-ZnO-2 (weight ratio CNT:ZnO = 1:10)—nanocomposite sample: (**a**) polyhedreral rod-like shaped ZnO nanoparticles covered with CNT (scale 100 nm); (**b**) SAED obtained on ZnO nanoparticles covered on the surface with CNT; (**c**) HR-TEM image of nanocomposite powder at 10 nm; (**d**) detail of MWCNT presented in (**c**,**e**) detail of crystalline ZnO presented in (**c**).

TEM images of CNT-ZnO-2 nanocomposite powder are shown in Figure 8a–e.

It can be observed thin chains of carbon nanotube on the surface of ZnO particles (Figure 8a,c). Polyhedral rod-like shaped ZnO nanoparticles have an estimated width between 24 and 80 nm and an approximate length around 39–160 nm. TEM results confirm our hypothesis regarding the existence of some interactions between ZnO and CNT and the formation of nanocomposites with a possible structure presented in Figure 2. Figure 8c shows CNT chains attached on the surface of ZnO nanoparticles through carboxylic groups of CNTs. During hydrothermal synthesis, this process of forming the physical bonds between the carboxylic groups of CNT and ZnO nanoparticles is favored by pressure. The carboxylic groups on the MWCNTs surface act as reaction sites in the hydrothermal process [9], leading to the formation of Zn-O-OOC-MWCNT structure presented in Equation (2), simultaneously with ZnO crystallization. From SAED Image presented in Figure 8b, we can identify only hexagonal ZnO. Insets presented in Figure 8d,e clearly shows the formation of MWCNT (Figure 8d) and highly crystalline ZnO nanoparticles, on which we can identify the 100 orientations of the Miller indices of 2.81 Å.

**Figure 9:**
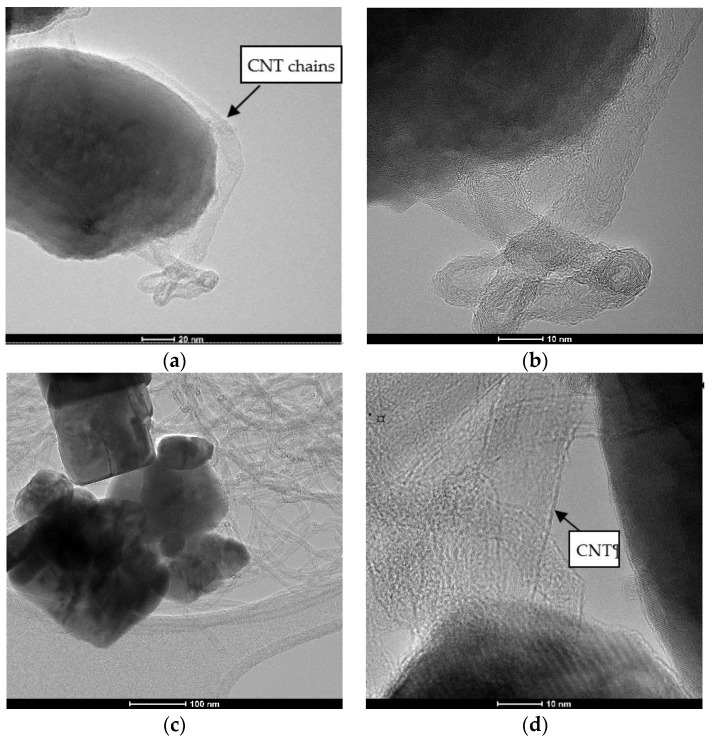
TEM images of CNT-ZnO-3 (weight ratio CNT:ZnO = 1:5) and CNT-ZnO-4 (weight ratio CNT:ZnO = 1:4) nanocomposite powders: (**a**) CNT-ZnO-3 sample. Polyhedreral rod-like shaped ZnO nanoparticles covered with CNT (scale 20 nm); (**b**) HR-TEM image of CNT-ZnO-3 nanocomposite powder at 10 nm; (**c**) CNT-ZnO-4 sample. Polyhedreral rod-like shaped ZnO nanoparticles covered with CNT (scale 100 nm); (**d**) HR-TEM image of CNT-ZnO-4 nanocomposite powder at 10 nm.

Figure 9 shows TEM images of samples CNT-ZnO-3 (weight ratio CNT:ZnO = 1:5) and CNT-ZNO-4 (weight ratio CNT:ZnO = 1:4). In the case of CNT-ZnO-3 nanopowder, it can be observed a ZnO particle covered by CNT chains (Figure 9a,b). However, when the percent of CNT increases in nanocomposite samples (CNT-ZnO-4), ZnO nanoparticles are entrapped in CNT mass. A high degree of agglomeration for CNT chains is observed. In the same time, CNT are attached on the surface of ZnO through carboxylic groups for all nanocomposite samples. As concluded also from thermal analysis (DSC-TG) and SEM characterization, it could be said that sample CNT-ZnO-2 (CNT:ZnO weight ratio = 1:10) is the most suitable from the point of view of physical interactions between the two components of nanocomposite, thermal behavior and morphological characteristics.

### 3.6. XRD Characterization of ZnO-CNT Nanocomposites

**Figure 10:**
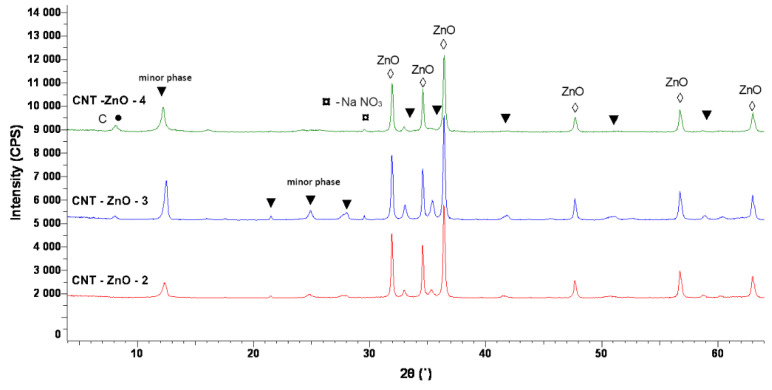
XRD spectra of CNT-ZnO-2 (1:10)—red; CNT-ZnO-3 (1:5)—blue, and CNT-ZnO-4 (1:4)—green nanocomposite samples.

X-ray diffraction spectra of ZnO-CNT nanocomposite samples are presented in Figure 10. Figure 10 presents the overlapped XRD patterns of the obtained samples. The XRD analysis revealed the presence of a major phase with hexagonal structure, which can be attributed to ZnO (ICDD PDF no. 01-070-8070). Moreover, the analysis reveals the existence of other minor phases, probably due to zinc sulphate appeared as a secondary product during CNT functionalization. Thus, a crystalline phase with a hexagonal symmetry (space group P3 (143), Pearson symbol hP46), (ICDD PDF no. 00-045-1386) is observed in all nanocomposite samples. C (from carbon nanotubes chains, (ICDD PDF no. 01-074-2328)) and NaNO_3_ (as the secondary reaction product of hydrothermal synthesis, according to equation 1, ICDD PDF no. 04-007-2328) structures could be detected in the case of CNT-ZnO-3 (CNT:ZnO = 1:5 mass ratio) and CNT-ZnO-4 samples (CNT:ZnO = 1:4 mass ratio). The presence of C (from CNT chains) in the XRD spectra of CNT-ZnO-3 and CNT-ZnO-4 samples demonstrates the excess amount of CNT in the nanocomposite samples, in accordance with the observations from thermal analysis results.

The crystallite size calculated in (101) direction, using Scherrer equation [6] (Equation (3)) is 61 nm in the case of CNT-ZnO-2 sample (CNT:ZnO = 1:10 mass ratio), around ~60 nm for CNT-ZnO-3 sample (CNT:ZnO = 1:5 mass ratio), and 54 nm in the case of CNT-ZnO-4 sample (CNT:ZnO = 1:5 mass ratio), decreasing slightly with the increase of CNT content.

(4) $$D=\frac{\kappa \lambda }{\beta cos\theta }$$

where:

*D*—the average crystallite size, in nm
*β*—the line broadening at half the maximum intensity, in radians,
*λ*—the X-ray wavelength, in Å;
*κ* = constant; *κ* = 0.9 according to Bragg or 0.70 < *κ* < 1.70 according to Klug and Alexander
*θ*—diffraction (Bragg) angle.

### 3.7. Extrusion-Based 3D Printing of ZnO-CNT Nanocomposite Powders

Based on the results obtained at characterization of nanocomposite powder, sample ZnO-CNT-2 having CNT:ZnO = 1:10 mass ratio has been chosen as a potential candidate to be processed in the form of pastes for preliminary experiments concerning fabrication of ZnO-CNT 3D structures by extrusion-based 3D printing.

**Figure 11:**
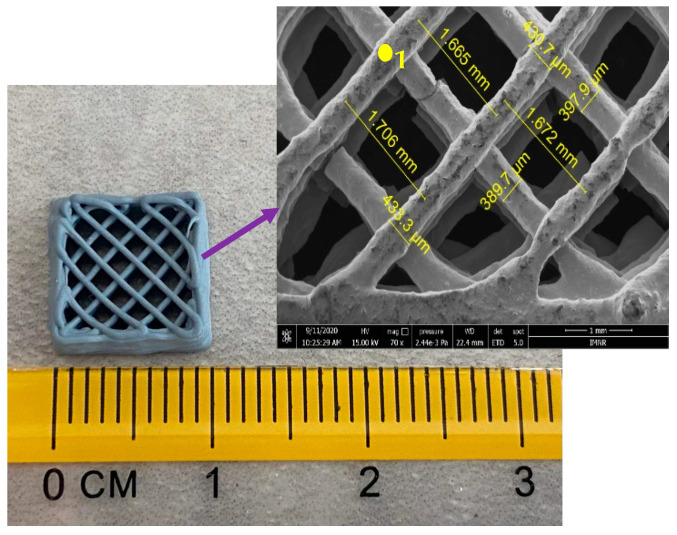
An example of CNT-ZnO 3D printed structure (3D-9 sample) at normal scale and microscopic scale.

In order to obtain the 3D structures from the ZnO-CNT nanocomposite powders several polymeric additives (binders, extrusion agents, viscosity agents) were tested: Mowiflex (crosslinked polyvinyl alcohol), Baymedix (aqueous polyurethane dispersion), poly (acrylic acid sodium salt) (PAAS), polyethyleneimine (PEI), ethylene glycol (EG), hydroxypropylmethylcellulose (HPMC), Tween 80 (polysorbate 80), Triton X-100 (polyoxyethylene octyl phenyl ether), and polylactic acid (PLA). The compositions of the prepared pastes are shown in Table 5. The additive type or the amount used significantly affects the printability and homogeneity of the obtained pastes. A homogeneous and printable paste was obtained by adding a combination of HPMC, PEI, and Tween 80 additives. The optimum composition that leads to a printable paste and to the tridimensional structure shown in Figure 11 was: 45.1% CNT-ZnO-2 nanopowder, 26.9% HPMC, 3.4% Tween 80, and 24.6% PEI. This composition corresponds to the structure 3D-9 in Table 5.

The 3D-9 sample is presented in Figure 11, emphasizing the dimensions of the 3D structure both on a normal scale and on a microscopic scale.

**Figure 12:**
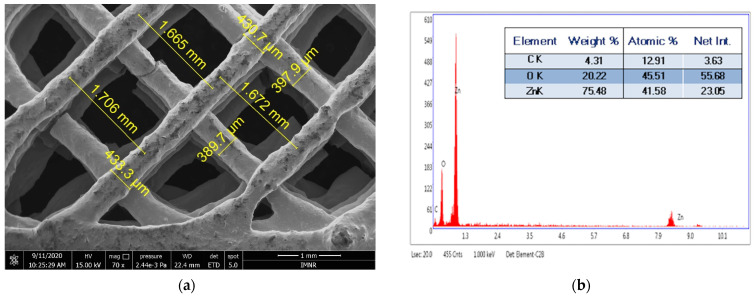
(**a**) SEM micrograph of 3D-9 sample; (**b**) EDS spectrum of 3D-9 sample.

SEM image of 3D-9 sample is depicted in Figure 12a. The strand thickness varies between 397.9 and 433.3 µm, in accordance with the nozzle diameter of 400 µm. The distance between fibers varies between 1.665 and 1.706 mm, in accordance with the selected distance of 2 mm. Results of the EDS analysis of the 3D-9 sample performed in spot 1 shown in Figure 12a are presented in Figure 12b. The existence of C, O, and Zn can be observed.

## 4. Conclusions

Different types of ZnO-CNT-based nanocomposite powders were obtained by hydrothermal process in high-pressure conditions, aiming to investigate the potential of CNT-ZnO nanocomposite powders to be processed in the form of pastes for 3D printing by extrusion. The formation of functionalized CNT-ZnO-nanocomposites has been demonstrated by spectral, thermal, and morpho-structural investigations. Thus, FT-IR spectra suggest the existence of some interactions between carboxylic groups of functionalized CNT and ZnO, by shifting or masking specific vibration bands of functionalized CNT. The presence of ZnO is confirmed by the specific vibration band of metal-oxygen bonds at 592 cm^−1^. DSC-TG thermal analysis confirms the formation of physical bonds between functionalized CNT and ZnO, by the appearance of some endothermic peaks in the range 196–233 °C, which could be due to the breaking of these CNT-ZnO bonds. TEM results clearly showed thin chains of CNT on the ZnO surface and the interaction between them, confirming the formation of nanocomposites with physical bonds in the case of CNT-ZnO-2 sample (CNT:ZnO mass ratio = 1:10).

Correlation of DSC-TG and XRD results suggested that an excess amount of CNT, which is not bonded with ZnO is present in CNT-ZnO-3 (CNT:ZnO mass ratio = 1:5) and CNT-ZnO-4 (CNT:ZnO mass ratio = 1:4) samples. Further studies for the fabrication of ZnO-CNT 3D structures are based on nanocomposite powders having CNT:ZnO mass ratio = 1:10 (CNT-ZnO-2 nanocomposite powder), which has been chosen as the most appropriate for the preparation of printable pastes. An example of CNT-ZnO 3D structure fabricated by extrusion-based 3D printing process, starting from hydrothermally synthesized nanocomposites and polymeric binders has been shown. Further work is under development to find some possible applications of ZnO-CNT 3D nanocomposite structures.

## Figures and Tables

**Table 1:** Representative samples of CNT functionalized by acid treatment.

Sample Name	Composition	Drying Method
CNTFAS-1	HNO_3_ 4 M:H_2_SO_4_ 10 M = 1:3	rotary evaporation
CNTFAS-5	HNO_3_ 2 M:H_2_SO_4_ 10 M = 1:3	freeze drying
CNTFAS-6	HNO_3_ 2 M:H_2_SO_4_ 5 M = 1:3	freeze drying

**Table 2:** Representative ZnO-CNT nanocomposite powders prepared by hydrothermal synthesis.

Nanocomposite Sample Code	Composition (Weight Ratio)
CNT-ZnO-2	CNT:ZnO = 1:10
CNT-ZnO-3	CNT:ZnO = 1:5
CNT-ZnO-4	CNT:ZnO = 1:4

**Table 3:** Thermal effects and mass loss (Δm) in acid-treated CNT powders.

Sample Name	Peak 1 (Endotherm)		Peak 2 (Endotherm)		Peak 3 (Endotherm)		Δm Total, %
	T, °C	ΔH, J/g	T, °C	ΔH, J/g	T, °C	ΔH, J/g	
CNTFAS-1					321.8	762.4	−94.865
CNTFAS-5	46.6	19.9	78.1	46.0			−44.211
CNTFAS-6	47.5	10.9	204.3	2.9			−17.416

**Table 4:** Thermal effects and mass loss (Δm) in ZnO-CNT nanocomposite powders.

Sample Name	Peak 1 (Endotherm)		Peak 2 (Endotherm)		Peak 3 (Endotherm)		Peak4 (Endotherm)		Δm Total, %	% CNT (Theoretic)
	T, °C	ΔH, J/g	T, °C	ΔH, J/g	T, °C	ΔH, J/g	T, °C	ΔH, J/g		
CNT-ZnO-2	63.2	20.4	233.7	75.4	-	-	631.7	26.3	9.7	9.1
CNT-ZnO-3	46.5	15.6	196.5 231.6	91.0 5.1	290.9	29.4	-	-	17.6	16.7
CNT-ZnO-4	55.2	18.6	231.5	65.3	326.3	2.1	-	-	18.4	20

**Table 5:** The composition of printable pastes used for the manufacture of 3D structures from CNT-ZnO-2 nanopowders.

No.	3D Object Name	Polymeric Additives	Printing Parameters	Observations
1	3D-1	Mowiflex 20% BAYMEDIX	-	Non-homogeneous paste. Non-printable paste.
2	3D-2	HPMC PAAS	-	The paste could not be extruded through the nozzle. Non-printable paste.
3	3D-3	HPMC PAAS	P = 5 bar, v = 3.5 mm/s nozzle φ = 0.4 mm	The paste is extruded in dots. The wire is not continuous. Non-printable paste.
4	3D-4	Tween 80 HPMC ethylene glycol	-	The paste is not homogeneous and is partially extrudable. Non-printable paste.
5	3D-5	HPMC PEI	P = 3.5 bar, v = 10 mm/s nozzle φ = 0.4 mm	Printable paste, a 3D object was obtained.
6	3D-6	HPMC PEI Tween 80	P = 3.5 bar, v = 10 mm/s nozzle φ = 0.4 mm	Printable paste, a 3D object was obtained.
7	3D-7	HPMC PEI Tween 80	P = 3.5 bar, v = 10 mm/s nozzle φ = 0.4 mm	Printable paste, a 3D object was obtained.
8	3D-8	HPMC PEI Tween 80	P = 2 bar, v = 15 mm/s nozzle φ = 0.4 mm	Printable paste, a 3D object was obtained.
9	3D-9	PEI HPMC Tween 80	P = 2 bar, v = 15 mm/s nozzle φ = 0.4 mm	Printable paste, a 3D object was obtained.
10	3D-10	PEI HPMC Tween 80	P = 1.5- 2 bar, v = 12- 16 mm/s nozzle φ = 0.4 mm	Printable paste, a 3D object was obtained.
11	3D-11	PEI Tween 80	P = 4.5 bar, v = 7 mm/s nozzle φ = 0.4 mm	Printable paste, a 3D object was obtained.
12	3D-12	PEI Tween 80	P = 4.1 bar, v = 6 mm/s nozzle φ = 0.4 mm	Printable paste, a 3D object was obtained.

## Data Availability

The data presented in this study are available on request from the corresponding author.
